# Dermal effects and pharmacokinetic evaluation of the lidocaine/prilocaine cream in healthy Chinese volunteers

**DOI:** 10.1186/s40360-023-00690-x

**Published:** 2023-10-12

**Authors:** Lingjun Li, Baole Cai, Hongyang Li, Jun Wei, Lei Tao, Pengcheng Ma

**Affiliations:** Hospital for Skin Diseases, Institute of Dermatology, Chinese Academy of Medical Sciences & Peking Union Medical College, Nanjing, 210042 Jiangsu China

**Keywords:** Dermal Effects, Pharmacokinetic Evaluation, EMLA

## Abstract

**Background:**

EMLA cream is a local anesthetic. The pharmacokinetics and dermal effects of a topical anesthetic formulation has not been evaluated in healthy Chinese volunteers.

**Materials and methods:**

The Pharmacokinetics of the lidocaine/prilocaine test (T) or reference (R, EMLA) cream were evaluated in a fasting, single-dose, two-period crossover bioequivalent study conducted in 40 healthy Chinese volunteers. Meanwhile, the dermal effects including blanching, erythema, temperature sensation, edema, and skin rash were also evaluated during the study.

**Results:**

After applied 15 g of the cream for 4 h to a 100 cm^2^ area under plastic occlusive film on the skin of the thigh of healthy volunteers, the results of the pharmacokinetic study showed that the active components absorbed in skin from topical products was relatively low compared with most system absorption drugs. After the removal of the residual anesthetic cream, there was a vascular biphasic response with initial transient blanching which reaches a peak at 4.5 h and later more persisting period erythema. The change of temperature sensory sensitivity reached the peak value at 4.5-6 h.There was no statistically significant difference of the changes after application the lidocaine/prilocaine T or R cream in subjects. In general, the lidocaine/prilocaine T or R cream was well tolerated.

**Conclusion:**

The method described a model for investigations of pharmacokinetics and pharmacodynamics of topical lidocaine/prilocaine cream. Except the plasma drug level indicator, these pharmacodynamics data should also be evaluated in the anesthetic transdermal pharmacokinetics study.

**Clinical Trial Registration:**

CTR20211544; registered in http://www.chinadrugtrials.org.cn/ at September 2021.

## Introduction

EMLA cream is a topical formulation containing 2.5% lidocaine and 2.5% prilocaine. The oil phase of the cream is a eutectic mixture of lidocaine and prilocaine in a ratio of 1:1 by weight. It is indicated as an amide-type local anesthetic that causes sensory nerve block by interfering with the passage of sodium ions through the membrane of nerve cells. It is used to produce local analgesia on normal intact skin or genital mucous membranes for superficial minor surgery after application under occlusive dressing [1].

The Pharmacokinetics of the topical of lidocaine/prilocaine test (T) or reference (R, EMLA) cream was evaluated in a fasting, single-dose, two-period crossover bioequivalent study conducted in 40 healthy Chinese volunteers. Meanwhile, the comparability of important local skin reactions for the test and reference products was evaluated during the study. Observations of local skin reactions including blanching, erythema, temperature sensation, edema, and skin rash were also noted [2]. This study also described the relationship of dermal effects and pharmacokinetics in healthy Chinese volunteers.

## Methods

### Ethics

The study was approved by the Ethics Committee of the Hospital of Dermatology, Chinese Academy of Medical Sciences & Peking Union Medical College (Nanjing, China) (ethics approval number: 2021-LC-001), according to the Declaration of Helsinki.

### Subjects

Forty healthy Chinese volunteers ( 27 males, 13 females; mean age 28.38 (18-44) years) participated in the study. Their mean body weight was 61.80 (49.10-76.10) kg; mean body mass index was 22.38 (19.70-26.00) kg/m^2^. After giving written informed consent form, a set of screen procedure in volunteers was conducted. All subjects should be in good health especially without any history of skin disease. Subjects with clinically significant abnormalities on physical examination, history of skin sensitive reaction against any topical drug or vehicle; currently receiving medication; routine laboratory tests (hematology, blood biochemistry, and urine analysis) or 12-lead electrocardiograms abnormal and women during breastfeeding or menstruating period were excluded.

### Drugs

2.5% Lidocainen / 2.5% prilocaine test (T) or reference (R) creams (EMLA) was supplied by Nanjing Changao Pharmaceutical Co. Ltd (Nanjing, China). The excipients of the cream include 2.5% lidocaine, 2.5% prilocaine, polyethylene glycol hydrogenated castor oil, carbomer and sodium hydroxide.

### Study design

The study was a fasting, single-dose, two-period crossover (T or R drug) design. The washout between two periods was one week. In the first period, after 10 h overnight fast, about 15 g lidocaine/prilocaine T or R cream was weighted on an occlusion film. Then the T or R cream was randomly administrated on a fixed area (10 × 10 cm^2^) on the skin of left thigh of the volunteers according to the random assignment and the applied site was covered by this occlusion film. The application time was 4 h.

Within 1 h before and after the dose application, water intake was forbidden. After application of the anesthetic, the occlusive dressing and the remaining product was removed with gentle toner.

The lunch and dinner were given to all subjects at 4 and 10 h after the dose application. Adverse events were monitored throughout the trial. Approximately 4 mL of blood was drawn into anticoagulant EDTA-K_2_ tubes at pre-dose (0) and 1, 2, 3, 3.5, 4, 4.5, 5, 5.5, 6, 6.5, 7, 7.5, 8, 8.5, 9, 9.5, 10, 11, 12, 16, 24, 36 and 48 h time point after dose application. The plasma samples were separated by being centrifuged at 2000 × g for 10 min and kept frozen at -60 °C until being LC-MS/MS analysis.

After one week of the period washout, the second procedure was the same as the first period, except the subject’s application site was on an equivalent area on the contralateral right thigh and the 15 g lidocaine/prilocaine test or reference cream was applied crossover.

### Analytical method

The analysis of the test was carried out in Jiangsu Wanlue Pharmaceutical Technology Co. Ltd. The plasma concentrations of lidocaine/prilocaine were measured using a validated liquid chromatography with tandem mass spectrometry (LC-MS/MS).The LC-MS/MS system was comprised of an Shimadzu LC-30AD HPLC analyzer (Shimadzu Technologies, Japan) and an API 5500 mass spectrometer (Applied Biosystems, USA) in the positive ion electrospray ionization-MRM(multiple-reaction monitoring) mode. Selected ion transitions were m/z 235.2→86.1 for lidocaine and m/z 221.2 →86.1 for prilocaine, respectively. The chromatographic separation was carried out on an ACE C_18_ column (50 mm × 2.1 mm) and the flow rate was set at 0.5 mL / min. The mobile phase was 0.4% formic acid (A) and methanol:acetonitrile:formic acid(6:94:0.4)(B). A gradient elution procedure was carried out in 3 min.

Lidocaine was detected in the concentration range of 0.1 to 50 ng/mL, while prilocaine was in the concentration range of 0.05 to 25 ng/mL, respectively. The intra- and inter-day precisions of the two analytes were all less than 15% (RSD, %).The mean recoveries of the three QC levels(low, medium, high) of lidocaine were 97.04%,91.93% and 88.98%, respectively, while the mean recoveries of prilocaine were 91.93%, 91.59% and 89.74%, respectively. The recoveries of two analytes were shown to be precise and reproducible in the plasma sample. The internal standard normalized matrix effect factor (IS-normalized MF) of lidocaine and prilocaine were all less than 15%. Stability experiments were carried out at two QC levels (low, high) during short-term storage (stored at room temperature, 1 and 2 h), after preparation storage (stored at 4 °C, 4 and 24 h), three freeze - thaw cycles (stored at -20 °C,-70 °C),and long-term storage (stored at -20 °C, -70 °C, 39 days). The results of the sample accuracy were all less than 15% which showed that two analytes were all stable during the preparation and analysis step.

### Plasma sample preparation

Fifty microliters of plasma sample was transferred to a 96-well plate. Twenty microliters of internal standards (5 ng/mL lidocaine-d10 hydrochloride, 2.5 ng/mL prilocaine- d7 hydrochloride) were added. Then the plasma samples were extracted by protein precipitation with 430 µL of acetonitrile. After being vortexed and centrifuged, the 100 µL supernatant was diluted with 100 µL 0.4% formic acid solution. Then an aliquot of 10 µL was injected for LC-MS analysis.

### Pharmacokinetics analysis

Phoenix Winnonlin software 8.2 was used to estimate the pharmacokinetics parameters of the non-compartment model, and the main pharmacokinetics parameters including T_max_, C_max_, AUC_0 - t_ and AUC_0-∞_ were calculated.

### Dermal effects assessment

After the removal of the plastic film occlusion and creams at 4 h, the skin reactions were examined and recorded including alteration in temperature sensation, blanching, erythema, edema and skin rash according to the grading criteria. The time point for evaluation was 4.5, 6, 12 and 24 h.

The temperature sensation was one of the indicators of the pharmacodynamics. The evaluation criteria are as follows:

-2 = Very cold sensation; -1 = cold sensation; 0 = no difference in temperature sensation; 1 = warm sensation; 2 = hot sensation.

Temperature sensation evaluation was conducted with two glass test tubes containing 5-10 °C and 40-45 °C water. The tubes were used to contact the skin of the non-applicated leg and the applicated leg, respectively. The subjects told the investigators the temperature sensation they felt. As a control, the skin temperature of the non-applicated leg was rated as -2 (very cold) or 2 (very hot). If the skin temperature did not reach the level of very cold or very hot, then the scores were - 1 (cold), 1 (hot) or 0 (no temperature sensation).The changes of the skin temperature sensation were obtained by subtracting the temperature score of the applicated leg from that of the non-applicated leg. The statistical differences of skin temperature sensation change between the lidocaine/prilocaine T and R preparations were investigated.

Because the degree of skin colour change after anesthetic application is correlated with the efficacy of the drug, this indicator can be used as an auxiliary observation index to judge the pharmacodynamics of the T and the R preparation (the main indicator is plasma drug concentration). The evaluation criteria of the skin tone changes are as follows:

+ 3 = Intense erythema (bright red, with or without petechia or papules); +2 = moderate erythema (pink-red, uniform over application site); +1 = mild erythema (faint-pink, uniform or spotty over application site); 0 = no change in skin tone; -1 = mild pallor (slight or indistinct outline of application site); -2 = moderate pallor (discernable outline of application site); -3 = intense pallor (clean distinct outline of application site).

The skin colour reaction evaluations ( blanching, erythema ) were conducted using simple naked-eye rated method. In order to reduce variability caused by the methodology, the colorimeter ( Konica Minolta CM-700d, Japan) was also used to standard the determination results. The results of the two methods will be analyzed and compared.

In addition, edema and skin rash were also observed in the clinical trial.

The evaluation criteria of edema and skin rash are as follows:

Edema:

0 = No edema; 1 = very slight edema, barely perceptible; 2 = slight edema; edges are well defined by definite raising; 3 = moderate edema, raised approximate 1 mm; 4 = severe edema raised more than 1 mm and/or extending beyond area of exposure.

Skin rash:

0 = No rash; 1 = mild rash (minor skin tone change, minimal edema, minimal popular response);2 = moderate rash (moderate skin tone change, marked papules, moderate or severe edema, vesicles);3 = severe rash (intense skin tone change, bullous or exudative eruptions, cracking, peeling, scabs, erosion, pustules).

### Statistical analysis

Data were analyzed by SPSS spearman test. P < 0.05 was considered significant.

## Results

### Pharmacokinetics

A total of 1872 blood samples were collected from the 40 subjects. The pharmacokinetic parameters were evaluated by using LC-MS/MS plasma drug concentration analysis method.

For lidocaine, the mean area under the plasma concentration-time curve (AUC_0 - t_) was 206.50 and 206.91 ng.h/mL, the mean maximum plasma concentrations (C_max_) of T and R cream were 22.0 and 22.9 ng/mL respectively and the achieved time (T_max_) was 8 h of both preparations.

**Fig. 1:**
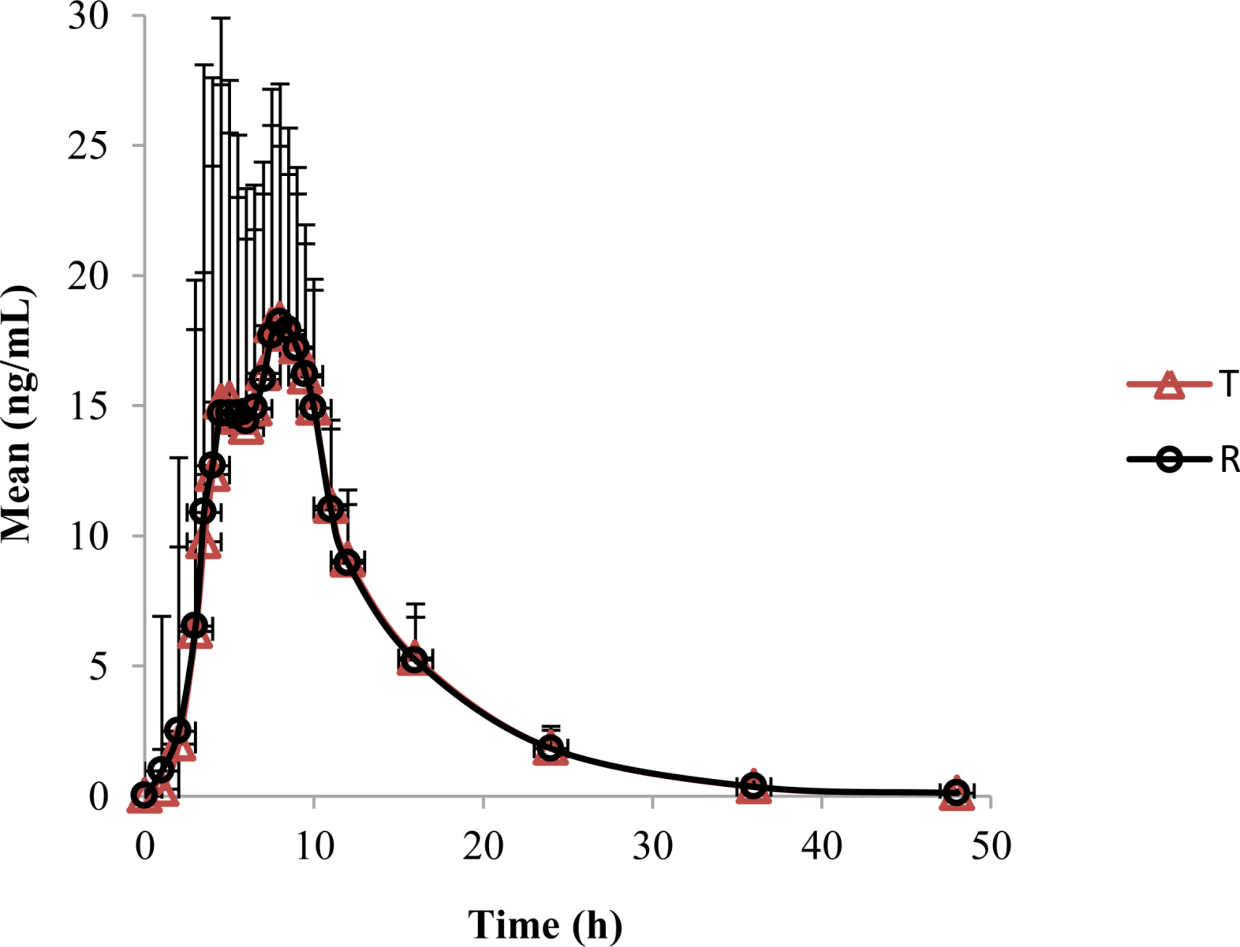
The mean plasma concentration-time curves of lidocaine

**Fig. 2:**
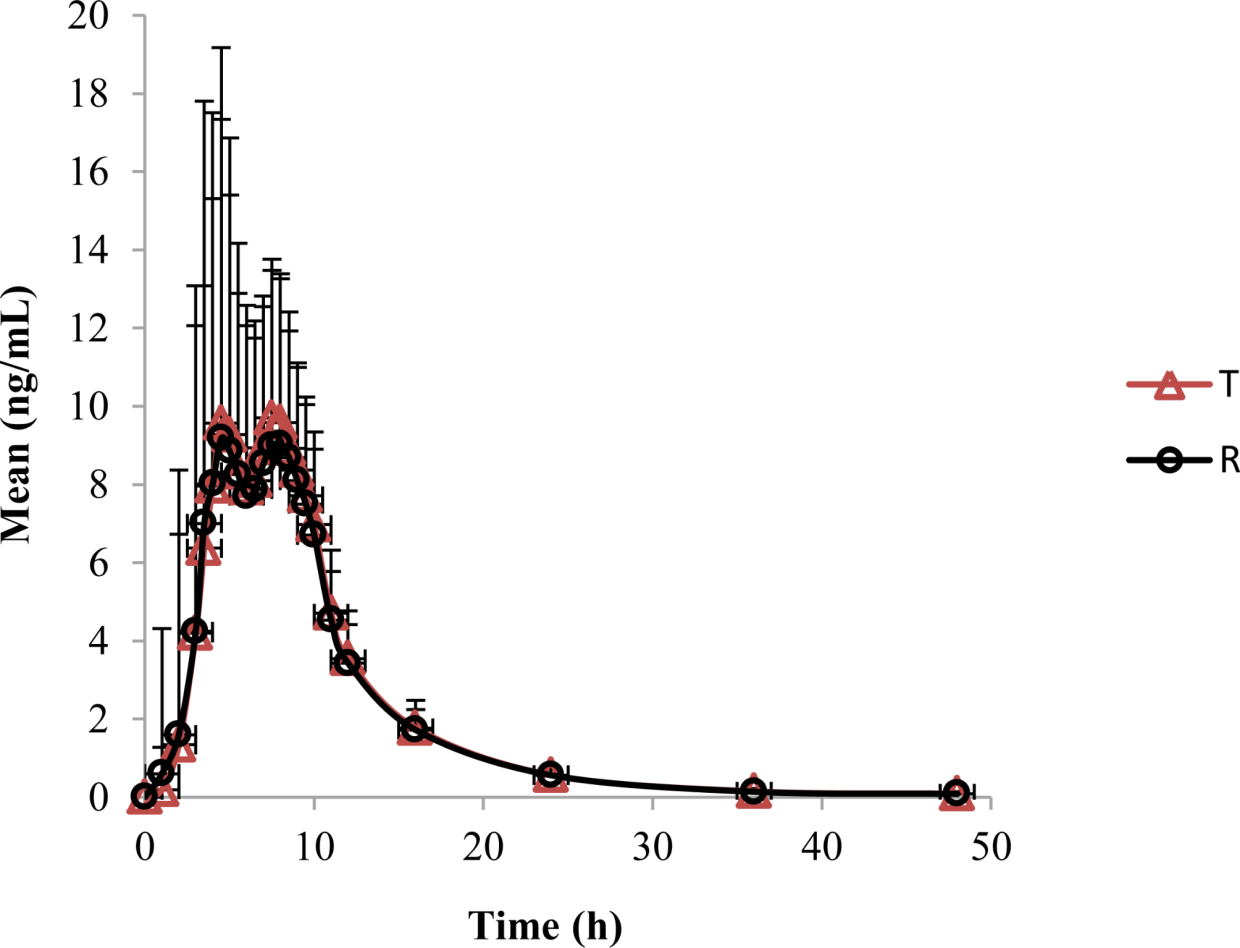
The mean plasma concentration-time curves of prilocaine

For prilocaine, the mean AUC_0 - t_ was 97.29 and 95.27 ng.h/mL, the mean C_max_ was 12.3 and 12.2 ng/mL, and the mean T_max_ was 7.5 and 8.0 h of T and R cream respectively. The mean plasma concentration-time curves of lidocaine and prilocaine were shown in Figs. 1 and 2.The mean pharmacokinetic parameters of lidocaine and prilocaine were summarized in Table 1.

**Table 1:** Mean pharmacokinetic parameters of lidocaine and prilocaine in T (n = 38) and R formulations (n = 40)

Parameter	Mean ± SD			
	lidocaine		prilocaine	
	T	R	T	R
C_max_ (ng/mL)	22.0 ± 10.9	22.9 ± 15.8	12.3 ± 7.0	12.2 ± 9.7
AUC_0 - t_(ng.h/mL)	206.5 ± 77.1	206.9 ± 95.7	97.3 ± 43.8	95.3 ± 55.8
AUC_0-∞_(ng.h/mL)	208.3 ± 77.1	208.6 ± 95.6	98.3 ± 43.6	96.2 ± 55.7
t_1/2_ (h)	5.6 ± 1.2	5.5 ± 1.2	6.5 ± 2.0	6.3 ± 1.7
T_max_^*^ (h)	8.0(3.5,12.0)	8.0(3.5,10.0)	7.5(3.0,10.0)	8.0(3.5,10.0)

*median (minimum, maximum)

### Pharmacodynamics

In the study, skin responses including blanching and erythema have been observed in skin areas treated with lidocaine/prilocaine cream.

**Fig. 3:**
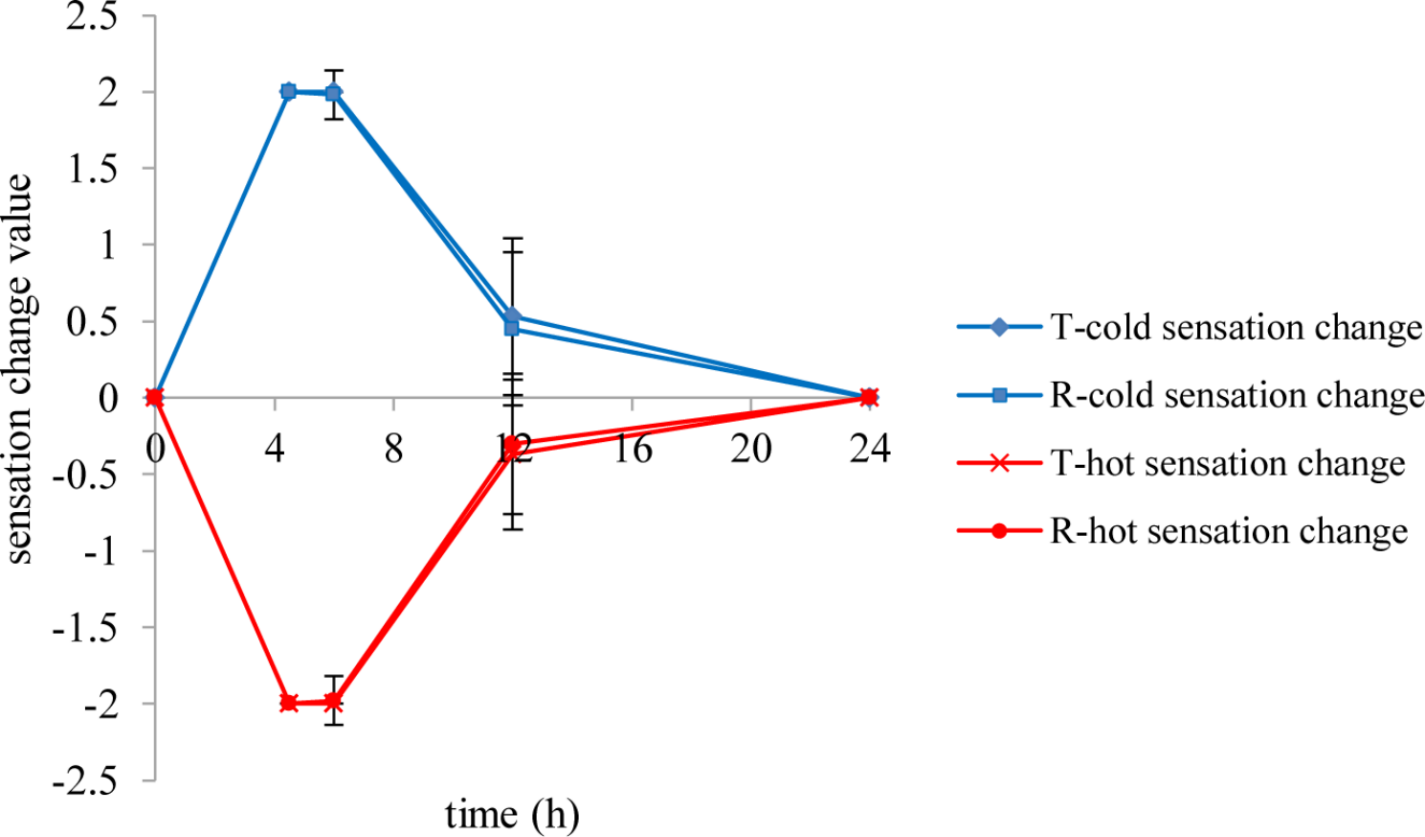
The cold and hot sensation change after application the lidocaine/prilocaine test or reference cream

The results of temperature sensation evaluation were shown in Fig. 3.

The change of cold and hot sensitivity reached the peak value at 4.5-6 h.There was no statistically significant difference of temperature sensation changes after application the lidocaine/prilocaine T or R cream in subjects (P > 0.05).

There was a vascular biphasic response after application of test or reference cream under plastic occlusive film with initial blanching and later erythema.

After four hour’s application of T cream on skin of thigh, blanching appearances were seen in 14/38 (36.8%) subjects at 4.5 h and the scores were all - 1 by using the naked eye observation. The blanching appearances were all disappeared at 6 h. Then the erythema appearances were observed in 31/38(81.6%) subjects at 4.5(8/38, 21.1%), 6(14/38, 36.8%), or 12 h (9/38, 23.7%) and faded away at 6-24 h.

While the blanching appearances of R cream were seen in 18/40 (45.0%) subjects at 4.5 h and the scores were - 1 in 39 subjects, only one subject’s score was - 2. In all cases, the blanching was very transient, vanishing at 6 h (the 2nd h after the end of application). The erythema appearances were seen in 34/40(85%) cases at 4.5(5/40, 12.5%), 6(21/40, 52.5%), or 12 h (8/40, 20.0%) and disappeared at 6-24 h.

**Fig. 4:**
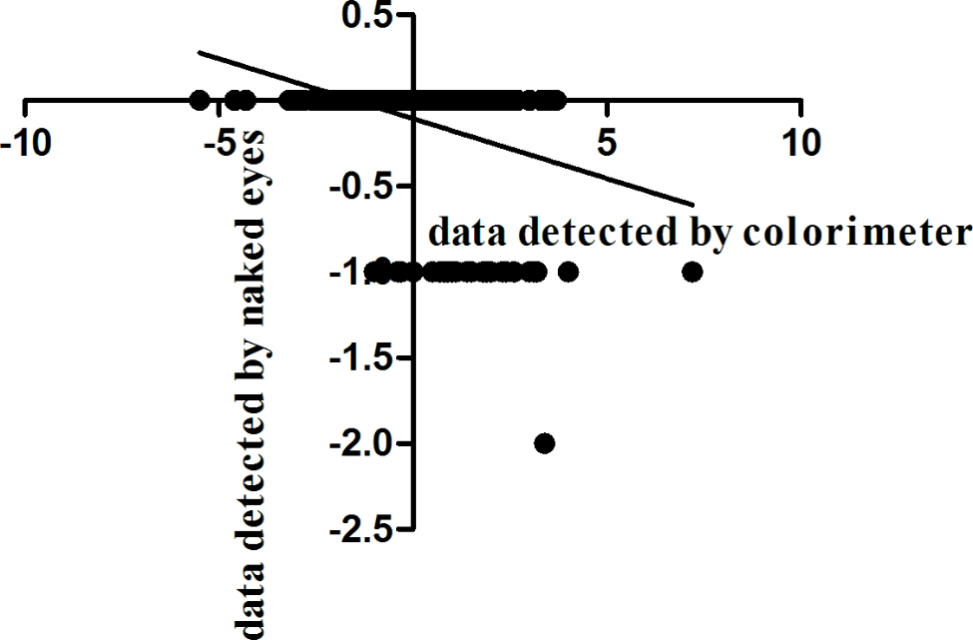
Spearman correlation test of the skin blanching reaction between the test or reference cream evaluated by colorimeter (x) and naked eyes (y)

**Fig. 5:**
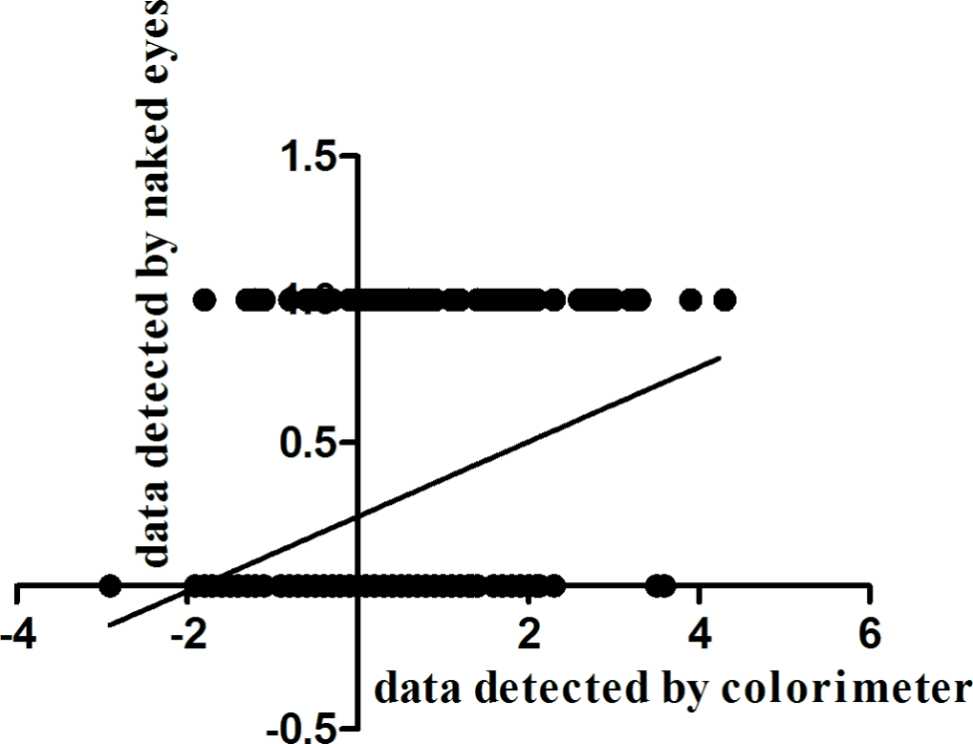
Spearman correlation test between the skin erythema reaction evaluated by colorimeter (x) and naked eyes (y)

The spearman correlation between the naked eye observation and the colorimeter was calculated by SPSS 18.0 software. The results of the correlation of the skin blanching and erythema reaction between the T or R cream evaluated by colorimeter and naked eyes were shown in Figs. 4 and 5. The spearman correlation coefficient was - 0.424 for the skin blanching reaction and 0.419 for the skin erythema reaction. The results of this study show that there was a correlation between the naked eye observation and the colorimeter evaluation methods. So the skin white and erythema could be measured by the instruments to get a more accurate value.

**Fig. 6:**
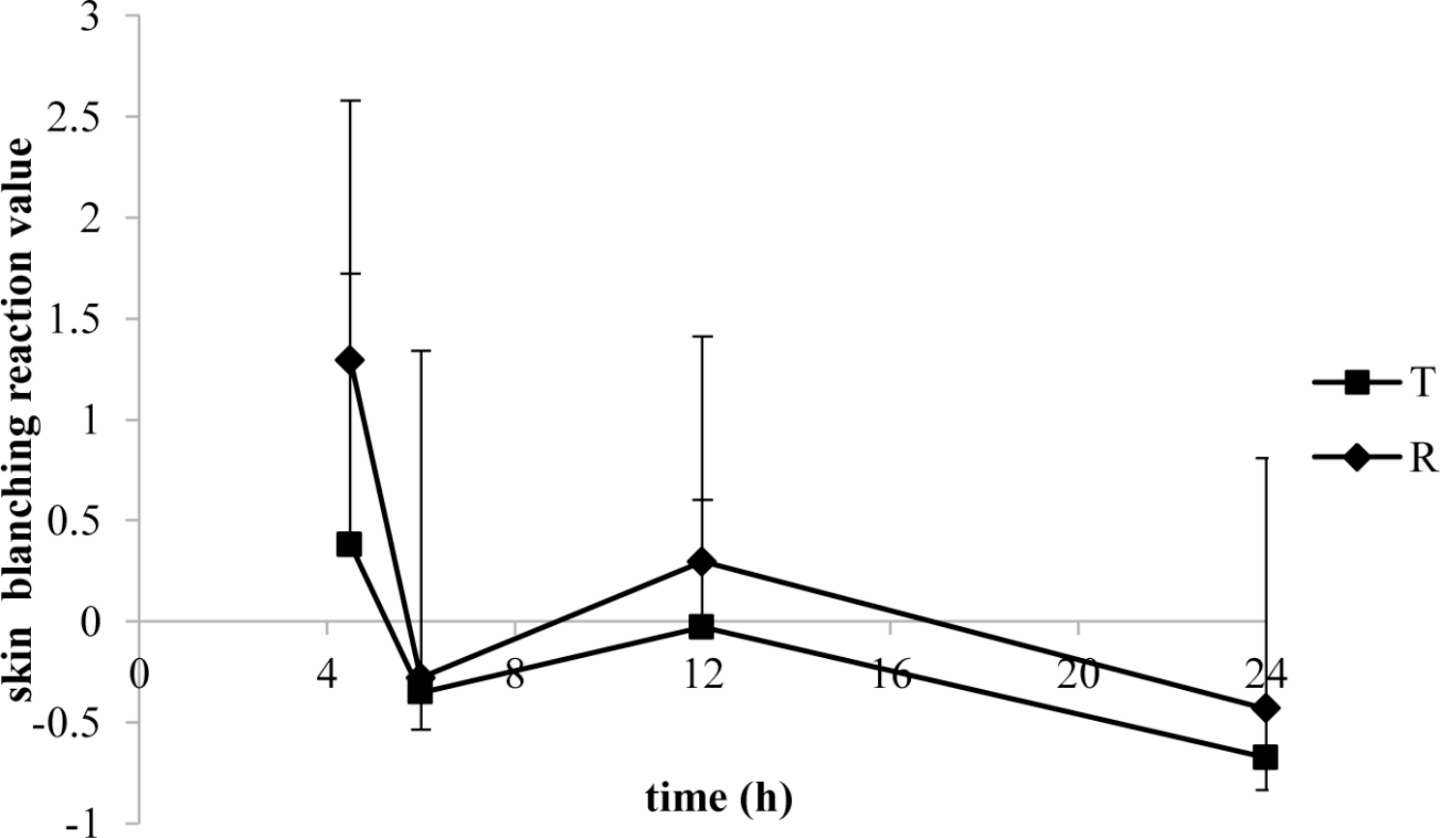
The skin blanching reaction values of lidocaine/prilocaine test or reference cream evaluated by colorimeter

**Fig. 7:**
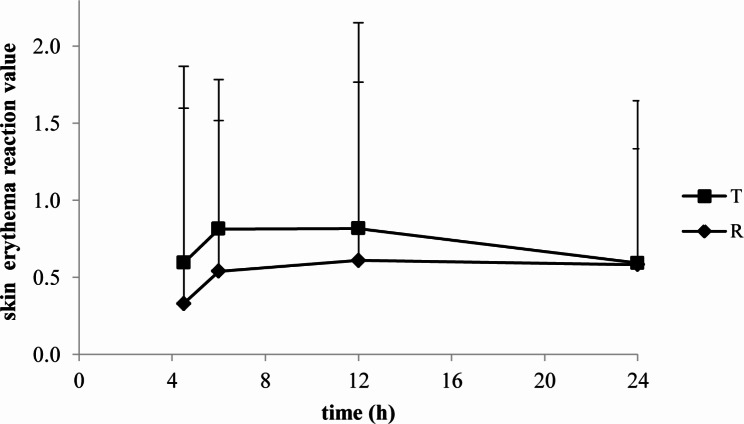
The skin erythema reaction values of lidocaine/prilocaine test or reference cream evaluated by colorimeter

The skin blanching and erythema reaction values of lidocaine/prilocaine T or R cream evaluated by colorimeter were seen in Figs. 6 and 7.

The skin blanching reaction values of T and R were both peaked at 4.5 h. The mean maximal erythema was obtained at 6-12 h (T) and 12 h (R) after a 4.5 h application. The erythema subsided within 24 h.

### Safety

In the study, the T and R creams were well tolerated by all participants. Only two subjects dropped out after completion of the first period for the adverse effects. One subject had infection of the upper respiratory tract and one affected hypersensitive C-reactive protein elevation. Both of the two adverse effects were not considered related to the drugs. No Edema, skin rash, or other skin reactions and no serious adverse events were reported by any of the subjects involved in this study.

## Discussion

This is the first study to evaluate the pharmacokinetics and dermal effects of a topical anesthetic formulation in healthy Chinese volunteers. Compared with most system absorption drugs, the active components absorbed in skin from topical products was relatively low. After applied 15 g of 2.5% lidocaine and prilocaine T or R cream for 4 h to a 100 cm^2^ area on the skin of the thigh of healthy volunteers, the results of the pharmacokinetic study showed that C_max_ for lidocaine were 22.0 and 22.9 ng/mL and AUC_0 - t_ was 206.5 and 206.9 ng.h/mL of T cream and R cream respectively. While the Mean C_max_ for prilocaine was 12.3 and 12.2 ng/mL and the mean AUC_0 - t_ was 97.3 and 95.3 ng.h/mL respectively. The values considered to be safe for both lidocaine and prilocaine. The maximal plasma levels occurred at 8 h for lidocaine and 7.5-8 h for prilocaine when the lidocaine and prilocaine creams were applied on the thigh skin.

For the property of corticosteroids to produce blanching or vasoconstriction in the microvasculature of the skin, the pharmacodynamics effect methodology, known as the vasoconstrictor assay, or the skin blanching assay has been developed and accepted by the Food and Drug Administration as a means of assessing potency and bioavailability of two potentially equivalent topical corticosteroid formulations [2, 3]. The colorimeter was recommended as a tool to qualify the degree of the skin blanching [3].

Just as the skin blanching property of topical corticosteroids, it has previously been reported that EMLA cream might also induce blanching of normal skin [4, 5]. So the comparability of local skin reactions including blanching, erythema, temperature sensation, edema, and skin rash for the T and R products were evaluated during the study. These reactions were observed at 4.5,6,12 and 24 h time points.

The study has shown that after application of lidocaine/prilocaine T or R cream under an occlusion dressing for 4 h, a blanching reaction was observed immediately without delay after removing the dressing and the residual products at 4.5 h.

The skin blanching appearances were observed in 14/38 (36.8%) subjects after application the T cream at 4.5 h and the scores were all - 1.While for the R cream, the skin blanching appearances occurrence incidence at 4.5 h were 18/40 (45.0%).The scores were - 1 in 39 subjects, only one subject’s score was - 2. The blanching appearances were all transient, reaching a peak at 4.5 h and disappearing in all cases 2 h after the end of application.

The initial skin blanching reaction was followed by an erythematous occurrence. An erythema was found in 31 cases out of 38 (81.6%) after application the T cream versus in 34 cases out of 40 (85%) after application the R cream. In 10 cases after application the T cream and 14 subjects after application the R cream, blanching and erythema were all shown with the initial blanching followed by erythema. Other subjects just occurred skin blanching (4/38, 10.5% for T cream and 4/40, 10.0% for R cream) or erythema (21/38, 55.3% for T cream and 20/40, 50.0% for R cream).Obviously, the incidence of erythema occurrence was much higher than that of skin blanching.

The erythema was all slight and the scores were all 1. The erythema appearances were observed in 31/38(81.6%) subjects for T cream at 4.5(8/38, 21.1%), 6(14/38, 36.8%), or 12 h (9/38, 23.7%) and faded away at 6-24 h. The erythema occurred in 34/40(85%) subjects for R cream at 4.5(5/40, 12.5%), 6(21/40, 52.5%), or 12 h (8/40, 20.0%) and disappeared at 6-24 h.Mean maximal erythema was obtained at 6-12 h (T) and 12 h (R). The erythema was all disappeared within 24 h.

Except the naked-eye observation method, the colorimeter method was also conducted to evaluate the skin colour reaction evaluations ( blanching, erythema). The values measured by the colorimeter are continuous changes, which can reflect the skin color changes more accurately, and reduce the systematic measurement error. The correlation analysis of the two methods showed that there was a correlation between the naked eye observation and the colorimeter.

In this study, the ‘biphasic kinetics’ of skin reactions was found. On average, skin blanching appeared and vanished earlier than skin erythema. That is to say: early occurrence of a transient blanching; later appearance of an erythema with a more persisting period. Skin erythema occurred after the end of blanching.

Just like the vasoconstriction property of topical corticosteroids in the skin vascular, both lidocaine and prilocaine can lead the cutaneous vasoconstrictive system for its local effect of the analgesics. This may be the main reason for the temporary skin blanching.

In addition, because the EMLA cream contains 95% water, skin occlusion may cause skin physiologic alterations in increasing stratum corneum hydration and promoting the uptake of water into intercellular lipid phase organization [6]. For the hydration of the stratum corneum, the optical scattering of the skin outer layers may increase the reflected light from the skin, which makes the skin surface colour white [7].

After the disappearing of the blanching, the later erythema following an initial blanching appeared. Why does an erythema occur later and last a more persisting period? After the occlusive dressing is removed, the stratum corneum dehydrates and becomes a reservoir, the local anesthetics are stored in the stratum corneum and the concentrations of local analgesics are increased slowly. At last, an intracutaneous depot of local analgesics is formed [8]. Except vasoconstrictive property, both lidocaine and prilocaine have concentration-dependent vasodilatative properties [9]. At lower concentrations, the local anesthetics firstly reach the sympathetic nervous system and lead to a vasoconstriction which cause skin blanching, while at higher concentrations, local analgesics reach the parasympathetic system [10]. Maybe the smooth muscle relaxant effect of the analgesics results a vasodilatation, so a following erythema appeared.

The temperature sensation inhibition effect can be used to evaluate the nerve sensory-blocking effect of transdermal local anesthetic [11]. A previous study demonstrated that duration of the sensory-blocking effect was different between lidocaine/prilocaine creams with different concentration [12]. Therefore, we speculate that the content and the concentration of topical lidocaine/prilocaine cream may affect the duration of nerve sensory block. In this study, there was no statistically significant difference of temperature sensation changes after application the lidocaine/prilocaine T or R cream in subjects (P > 0.5). The peak time of temperature sensation was 4.5-6 h, which was generally consistent with the achieved time of maximum plasma concentrations. We think that the temperature sensation inhibition effect can be used as a pharmacodynamics index to evaluate the bioequivalence of lidocaine/prilocaine T or R cream.

In general, the lidocaine/prilocaine T or R cream was well tolerated. Except skin blanching and erythema, there were no edema, skin rash, or other skin reactions and no serious adverse events reported in this study.

## Conclusions

The method described a pharmacokinetics and pharmacodynamics study method of topical lidocaine/prilocaine cream. It is useful in studies evaluating skin absorption of topical anesthetics. Except the plasma drug level indicator, these pharmacodynamics data should also be evaluated in the anesthetic transdermal pharmacokinetics study. By correlating the plasma concentrations with the local pharmacodynamics effects of the anesthetic, the method might also be used as a model for investigations of the transdermal characteristic of local anesthetic.

## Data Availability

The datasets generated and/or analysed during the current study are not publicly available due data confidentiality but are available from the corresponding author on reasonable request.

## Declarations

All volunteers provided written informed consent forms before participation in the study and after having been fully informed about the aim, course, and possible risks of the study.
